# Factors Influencing Dropout and Retention Among Autistic Students in Universities: A Meta-Synthesis of Qualitative Studies Involving Autistic Individuals

**DOI:** 10.1192/bjo.2025.10228

**Published:** 2025-06-20

**Authors:** Oriyomi Shittu, Georgia Smith, Megan Freeth

**Affiliations:** 1 Sheffield Health and Social Care NHS Foundation Trust, Sheffield, United Kingdom; 2 University of Sheffield, Sheffield, United Kingdom

## Abstract

**Aims:** Approximately 2% of students in higher education are diagnosed with autism, a figure likely underreported due to non-disclosure and diagnostic challenges. Autistic students in higher education face unique challenges that impact their academic persistence and success. These students experience higher dropout rates compared with their neurotypical peers. Identifying the factors leading to high dropout rates is essential for developing interventions that promote a more supportive academic environment for autistic students.

This review systematically investigates and analyses the factors that influence dropout rates among autistic students in higher education, focusing on firsthand accounts of autistic students. It draws on qualitative and mixed-method studies to address the question: What are the key factors influencing dropout rates among autistic university students across various degree programmes and institutions worldwide? The aim is to identify insights that can inform interventions to improve educational outcomes for autistic adults.

**Methods:** This meta-synthesis, adhering to PRISMA guidelines, utilised the PICOS framework for a systematic review conducted in July 2024. Searches across Scopus, PsychINFO, Medline via OVID, and ERIC yielded 2,303 initial articles. Thirteen studies were selected based on relevance and peer review, involving 521 participants aged 18–54, primarily diagnosed with autism spectrum disorder and based in the UK, USA, and Australia. All participants had either considered dropping out, actually dropped out, or deferred a year. These studies examined dropout rates and potential contributory factors, with findings appraised for quality using the CASP checklist and determined to be of moderate to good quality.

**Results:** Analysis of the thirteen studies found significant challenges faced by autistic students in higher education, revealing an overarching theme of 'Environmental Discord’ – systemic mismatches between educational structures and autistic students’ needs that exacerbate academic and mental health challenges. Mental health emerged as a critical factor influencing dropout rates with exacerbated conditions such as higher burnouts, anxiety and depression due to academic pressures and social isolation. Additionally, the studies highlighted academic challenges that hinder effective learning and engagement. The analysis also identified gaps in support strategies, indicating that existing services often fail to meet the specific needs of autistic students, necessitating tailored interventions.

**Conclusion:** This review proposes proactive restructuring of educational environments to better accommodate diverse student needs, enhance success and reduce dropout rates. Further, the review calls for future research to develop and evaluate personalised interventions aimed at the specific challenges faced by autistic students.

